# Reusing and/or reprocessing the N95 face respirator mask or equivalent: An integrative review

**DOI:** 10.1590/1518-8345.5135.3492

**Published:** 2021-10-29

**Authors:** Elucir Gir, Mayra Gonçalves Menegueti, Laelson Rochelle Milanês Sousa, Natália Maria Vieira Pereira-Caldeira, Milton Jorge de Carvalho, Renata Karina Reis

**Affiliations:** 1 Universidade de São Paulo, Escola de Enfermagem de Ribeirão Preto, PAHO/WHO Collaborating Centre for Nursing Research Development, Ribeirão Preto, SP, Brazil.; 2 Centro Universitário Saúde ABC, Faculdade de Medicina do ABC, Santo André, SP, Brazil.

**Keywords:** Personal Protective Equipment, Pandemics, Coronavirus Infections, Facial Masks, Respiratory Protective Devices, Review, Equipo de Protección Personal, Pandemias, Infecciones por Coronavirus, Máscaras Faciales, Dispositivos de Protección Respiratoria, Revisión, Equipamento de Proteção Individual, Pandemias, Infecções por Coronavírus, Máscaras Faciais, Dispositivos de Proteção Respiratória, Revisão

## Abstract

**Objective:**

to analyze the scientific evidence available on the different reprocessing methods and the necessary conditions for reuse of the N95 face respirator mask or equivalent.

**Method:**

an integrative literature review. The PICO strategy was used to elaborate the question. The search was conducted in four databases: PubMed, SciVerse Scopus, WebofScience and EMBASE, considering any period of time.

**Results:**

a total of 32 studies were included from the 561 studies identified, and they were presented in two categories: “Conditions for reuse” and “Reprocessing the masks”. Of the evaluated research studies, seven(21.8%) addressed the reuse of the N95 face respirator mask or equivalent and 25(78.1%) evaluated different reprocessing methods, namely: ultraviolet germicidal irradiation(14); hydrogen peroxide(8); vapor methods(14); using dry heat(5) and chemical methods(sodium hypochlorite[6], ethanol[4] and sodium chloride with sodium bicarbonate and dimethyldioxirane[1]). We emphasize that different methods were used in one same article.

**Conclusion:**

no evidence was found to support safe reprocessing of face respirator masks. In addition, reuse is contraindicated due to the risk of self-contamination and inadequate sealing.

## Abstract (es)

**Objetivo:**

analizar la evidencia científica disponible sobre los diferentes métodos de reprocesamiento y las condiciones necesarias para la reutilización de una mascarilla respiratoria facial N95 o equivalente.

**Método:**

revisión integradora de la literatura. Para elaborar la pregunta se utilizó la estrategia PICO. La búsqueda se realizó en cuatro bases de datos PubMed, *Sci Verse Scopus, Web of Science* y EMBASE sin límite de tiempo.

**Resultados:**

de los 561 estudios identificados 32 fueron incluidos y presentados en dos categorías: “condiciones de reutilización” y “reprocesamiento de mascarillas”. De las investigaciones evaluadas, siete (21,8%) abordaron la reutilización de la mascarilla respiratoria facial N95 o equivalente y 25 (78,1%) evaluaron diferentes métodos de reprocesamiento: irradiación germicida ultravioleta (14); peróxido de hidrógeno (8); métodos de vapor (14); uso de calor seco (5) y métodos químicos hipoclorito de sodio (6), etanol (4) y cloruro de sodio con bicarbonato de sodio y dimetildioxirano (1). Cabe destacar que en un mismo artículo se utilizaron métodos diferentes.

**Conclusión:**

no se encontró evidencia que apoye el reprocesamiento seguro de las mascarillas respiratorias. Además, la reutilización está contraindicada debido al riesgo de autocontaminación y sellado inadecuado.

## Abstract (pt)

**Objetivo:**

analisar as evidências científicas disponíveis sobre os diferentes métodos de reprocessamento e as condições necessárias para reuso de máscara respiratória facial do tipo N95 ou equivalente.

**Método:**

revisão integrativa da literatura. Para elaboração da questão foi utilizada a estratégia PICO. A busca ocorreu em quatro bases de dados PubMed, *Sci Verse Scopus, Web of Science* e EMBASE considerando qualquer período de tempo.

**Resultados:**

foram incluídos 32 estudos dos 561 identificados e apresentados em duas categorias: “condições para reuso” e “reprocessamento das máscaras”. Das pesquisas avaliadas, sete (21,8%) abordaram o reuso da máscara respiratória facial do tipo N95 ou equivalente e 25 (78,1%) avaliaram diferentes métodos de reprocessamento: irradiação germicida ultravioleta (14); peróxido de hidrogênio (8); métodos a vapor (14); utilização do calor seco (5) e métodos químicos (hipoclorito de sódio (6), etanol (4) e cloreto de sódio com bicarbonato de sódio e dimetildioxirano (1). Destacamos que um mesmo artigo utilizou diferentes métodos.

**Conclusão:**

não foram encontradas evidências que sustentam o reprocessamento seguro de máscaras respiratórias faciais. Ainda, o reuso é contraindiciado devido ao risco de autocontaminação e vedação inadequada.

## Introduction

The world faces a pandemic regarded as the biggest health problem of the 21^st^century. The first cases of the disease due to coronavirus 2019(COVID-19), caused by coronavirus2 of the Severe Acute Respiratory Syndrome(SARS-CoV-2) were reported at the end of2019 in China^(1)^. The unprecedented spread of SARS-CoV-2 led to the declaration of a pandemic by the World Health Organization in March2020^(2)^. In just over a year, until June8^th^,2021, there were 173,271,769 confirmed cases worldwide, 3,733,980 deaths and 1,900,955,505 vaccine doses were administered^(3)^.

The Americas region experienced a rapid increase in the number of COVID-19^(3)^ reported cases. In Brazil, the first cases began in February2020^(4)^. Since then, the pandemic has so advanced in the country, computing 16,947,062 confirmed cases and 473,404 deaths by June8^th^,2021^(3)^, becoming the third country with the highest number of cases and the second in deaths in the world^(3)^.

High rates of infection caused harms in the health systems around the world, collapsing many of them^(5)^. Given this global problem, protection of the health professionals engaged in combating and controlling the pandemic emerges as a core issue, as they are at high risk for infection^(6)^. The spread of COVID-19 in the health services is worrying, with health professionals representing a disproportionately high percentage of the confirmed cases^(7)^.

An epidemiological study conducted in Brazil from March to May2020 identified 17,414 suspected cases, 5,732 confirmed cases and 134 deaths in Nursing professionals^(8)^.

Data from the Pan American Health Organization of September2^nd^,2020, indicate that approximately 570,000 health workers were infected and that 2,500 died due to COVID-19 in the Americas^(9)^.

For the safety of these professionals, it is necessary to ensure policies and best practices that minimize exposure to respiratory pathogens, including SARS-CoV-2, ensuring sufficient and good quality Personal Protective Equipment(PPE). However, the pandemic caused by SARS-CoV-2 resulted in a global shortage of PPE, including face respirator masks(FRMs)^(10)^. As the need for FRMs has increased on a global scale, prices and demand have significantly gone up to the point that many health institutions are unable to replenish their inventories.

In fact, with the advent of the COVID-19 pandemic, the supply of FRMs was compromised in many countries. Lack of PPE or the use of unsuitable materials for patient care has been reported by health professionals from all the Brazilian regions^(11)^. Given this crisis, when lack of PPE cannot be solved by reducing their use or increasing production^(8)^, the WHO has recommended measures for the rational use of PPE in the health services^(12)^.

According to this organization, the global stock of PPE is insufficient, given the global demand not only due to the number of COVID-19 cases, but also due to disinformation and panic buying and stocking, which aggravates the global shortage of PPE, especially for respiratory protective masks with a minimum particular filtration efficiency of95%, such as the N95 type or equivalent^(12)^.

The global shortage of FRMs led the health centers around the world to extend the use of these masks, although they were designed for single use^(13)^. In addition, the persistence and infectiousness of the infectious agents in the FRMs, such as the pandemic influenzaA virus(H1N1)^(14)^, other coronaviruses^(15)^ and more recently SARS-CoV-2, show the importance of developing guidelines and protocols related to decontamination of this PPE and stress the importance of proper handling of personal protective equipment during and after use in high-risk environments to minimize the probability of transmission by fomite^(16)^.

While there is no recommendation for reprocessing and reusing FRMs, such as N95 or equivalent, as a routine standard of conventional care, these measures may be needed during periods of scarcity to ensure continuous availability during a pandemic. However, it is noted that, for reprocessing FRMs, it is fundamental that the method is effective and able to reduce the load of pathogens, that it preserves the function of the face mask, and that it does not present any residual chemical risk^(17)^.

In Brazil, and in the face of the COVID-19 pandemic, the National Health Surveillance Agency(*Agência Nacional de Vigilância Sanitária*,ANVISA) recommends that the health institutions are to establish their protocols on the use of PPE based on the exposure risks(for example:type of activity) and on the dynamics of pathogen transmission(for example:contact, droplet or aerosol). As for the N95 or equivalent masks, ANVISA has instructed the health professionals to use them for a longer period of time than that indicated by the manufacturers, as long as the mask is intact, clean and dry; and it points out that such an indication is required, as many professionals are reporting low inventories to treat critically-ill patients in the Intensive Care Unit^(18)^.

The search for solutions to meet the challenge of scarcity of FRMs is urgent^(19)^. In the literature, there is a variety of potential disinfection methods for FRMs, such as: (1)energy methods(for example:dry and moist ultraviolet heat and microwave-generated vapor) or (2)chemical methods(for example:alcohol, ethylene oxide, bleach and vaporized hydrogen peroxide)^(20-21)^ and some methods, such as ultraviolet germicidal irradiation, hydrogen peroxide vapor and moist heat, have been regarded as promising^(17)^, while others such as alcohol and ultraviolet light cause functional degradation in different degrees in the FRMs^(20)^.

Given this context, the need is evidenced for a comprehensive literature review to identify the evidence on the safe methods for reprocessing and evidence that support or not reuse of N95 or equivalent masks.

## Objective

To analyze the scientific evidence available on the different reprocessing methods and the necessary conditions for reuse of N95 face respirator masks or equivalent.

## Method

### Type of study

An integrative literature review developed in accordance with the following stages: selection of the review question; sampling(search for studies according to the inclusion and exclusion criteria); extraction of the characteristics of the primary research studies(data extraction); data analysis; interpretation of the results; and review report^(22)^.

In addition, the recommendations of the *Preferred Reporting Items for Systematic Reviews and Meta-Analyses*(PRISM)^(23)^ were followed. Registration on the *Fig Email* platform was made, with DOI:https://doi.org/10.6084/m9.figshare.14515251

The PICO strategy^(24)^ was used to outline the guiding question, where: P(Patient/Population):N95 face respirator mask or equivalent, I(Intervention): reuse or reprocessing of N95 face respirator masks or equivalent, C(Comparison):Not applicable, O(Outcomes): Necessary conditions for reuse and reprocessing methods indicated for the N95 mask or equivalent, giving rise to the following guiding question: Which is the scientific evidence available on the different reprocessing methods and the necessary conditions for reusing N95 face respirator masks or equivalent?

### Selection criteria

The criterion for inclusion and selection of the studies was based on research studies that used some method to reuse and/or reprocess N95 face respirator masks or equivalent. There was no language restriction. The reprocessing techniques do not necessarily need to test the SARS-CoV-2 microorganism to be a potential method for reprocessing masks. It was for this reason that we decided not to limit the time for the search and not to restrict the search only to tests with SARS-CoV-2.

### Data collection

The search for the studies occurred by peers in June2020, in the PubMed(US National Library of Medicine), Scopus, WebofScience and EMBASE databases by using controlled descriptors and keywords with the aid of boolean operators AND and OR. The search strategy used for all databases was [(“Respiratory Protective Devices” OR “N95 respirator” OR “N95 mask” OR “filtering facepiece respirator” OR “FFP2” OR “PPE”) AND (“reprocessing” OR “reuse” OR “decontamination” OR “disinfection” OR “disinfection” OR “sterilization”)].

The search results were inserted into the Ayres web application for selecting the studies. Two researchers read the titles and abstracts and selected the articles. Disagreements related to selection were resolved by a third reviewer. Subsequently, full-reading of the articles selected in the first stage was also carried out by two reviewers. A third reviewer assessed the disagreements of the articles included. Consensus meetings were held in two stages.

For evaluating the evidence level of the studies, the methodological design of each of them was considered and, as all the descriptive studies addressed clinical issues on intervention/treatment or diagnosis/diagnostic test, the classification used was that of seven levels, as follows: LevelI-Evidence from systematic reviews or meta-analyses of multiple controlled clinical and randomized studies; LevelII-Evidence from at least one well-designed randomized controlled clinical trial; LevelIII-Evidence from well-designed non-randomized clinical trials; LevelIV-Evidence from well-designed cohort and case-control studies; LevelV-Evidence from systematic reviews using descriptive and qualitative methodologies; LevelVI-Evidence from only one descriptive or qualitative study; LevelVII-Evidence from concepts of authorities and/or expert committees’ reports^(25)^.

### Data extraction

The articles involved in the analysis had their information extracted with the aid of a proposed roadmap^(26)^, determining the main data to be extracted. In this study, the following information was extracted: title; year of publication; reuse/reprocessing; method employed in reprocessing; authors’ recommendations and level of evidence of the studies.

Data synthesis was descriptive. The reuse and reprocessing conditions identified were analyzed, grouped and compared. In this stage, two independent reviewers were responsible for extracting, analyzing and synthesizing the information.

## Results

**Figure 1:**
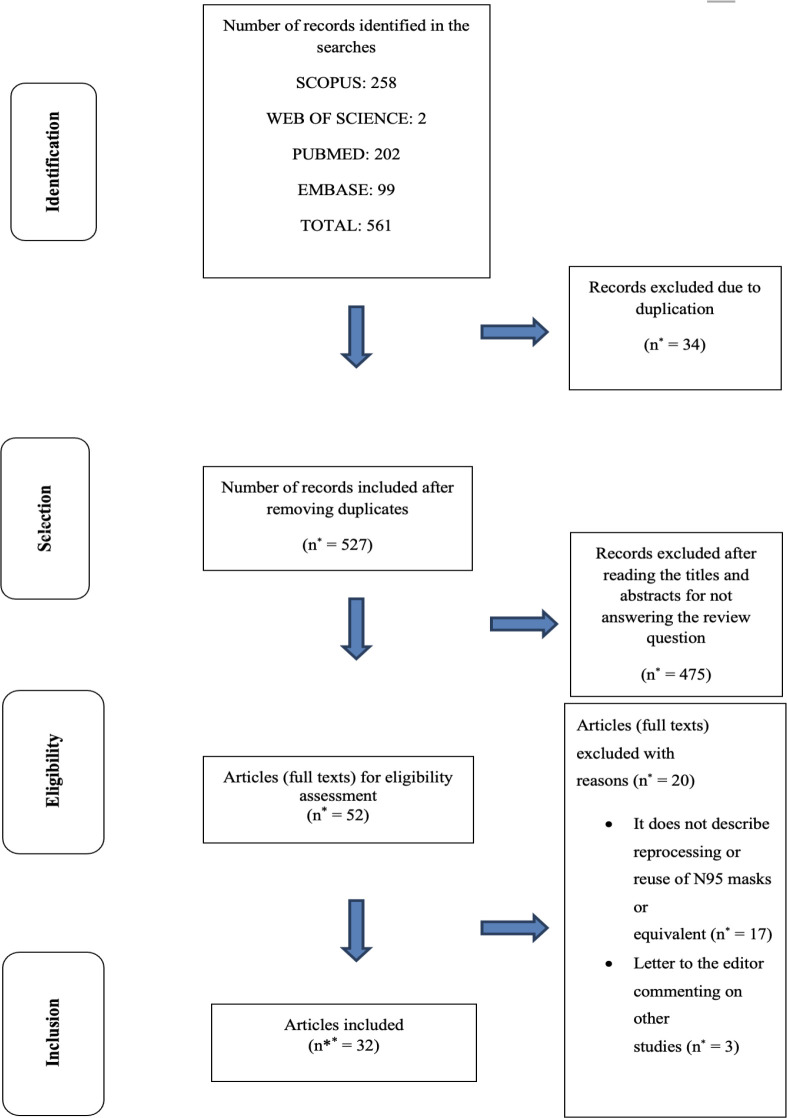
Diagram corresponding to the search and selection of the studies according to PRISMA^(23)^ *n=number of articles

Selection of the studies followed the PRISMA^(23)^ recommendations(Figure 1).

**Figure 2:**
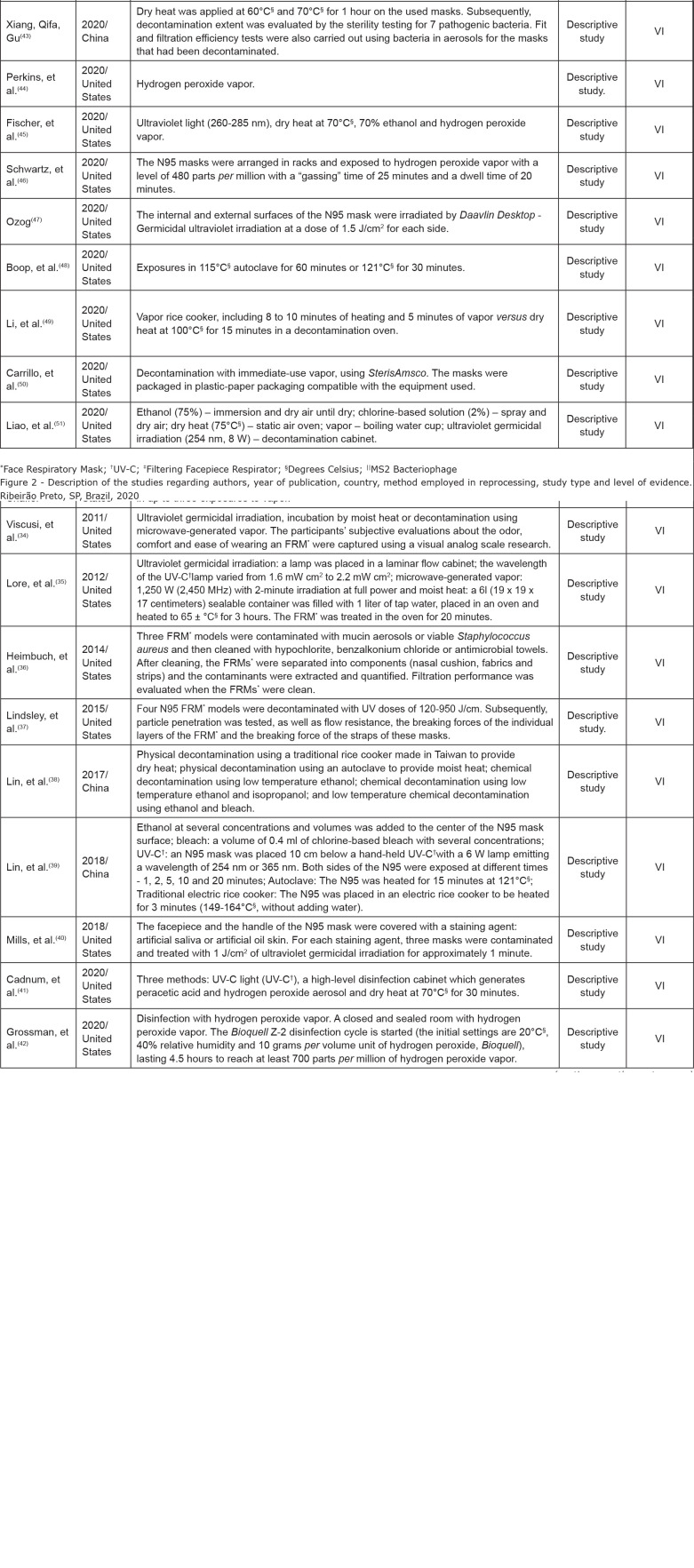
Description of the studies regarding authors, year of publication, country, method employed in reprocessing, study type and level of evidence. Ribeirão Preto, SP, Brazil, 2020

A total of 32 studies that evaluated reuse and reprocessing of N95 respirator masks or equivalent were included, with most of the studies being conducted in the United States(26=81.3%). The level of evidence was mostlyVI(25=78.1%). As for the language of the articles, 31(96.9%) were in English. Figure 2 shows the characteristics of the studies according to the authors, year of publication/country, method employed in reprocessing, study type and level of evidence.

**Figure 3:**
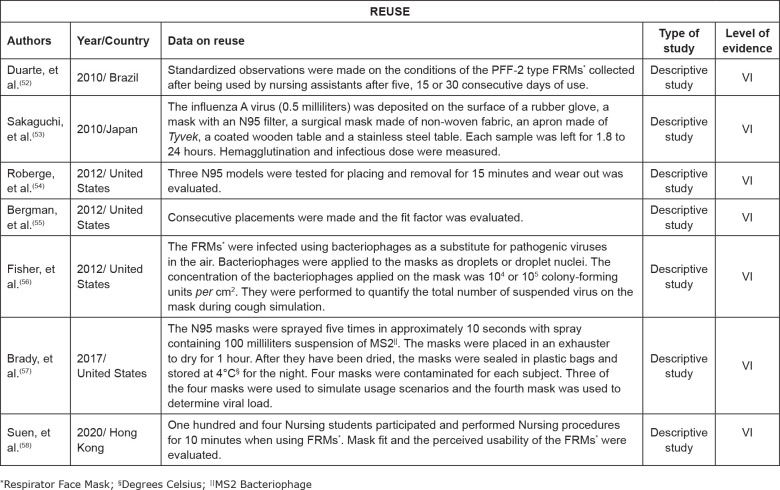
Description of the studies regarding authors, year of publication/country, reuse on data, type of study and level of evidence. Ribeirão Preto, SP, Brazil, 2020

Figure 3 lists the data of the authors, year of publication/country, data on reuse, type of study and level of evidence.

**Figure 4:**
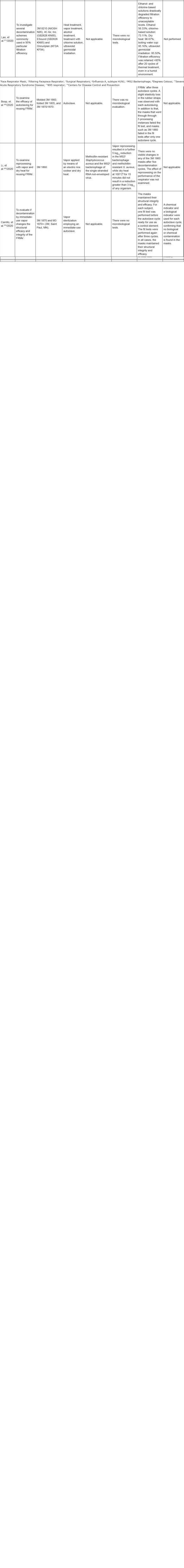
Description of the studies regarding authors, objectives, type of mask, reprocessing method, type and size of the microorganisms, efficacy of each type of reprocessing, effect of reprocessing on the structure of the masks and chemical risk. Ribeirão Preto, SP, Brazil, 2020

Descriptions of the studies regarding authors, objectives, type of mask, reprocessing method, type and size of the microorganisms, efficacy of each type of reprocessing, effect of reprocessing on the structure of the masks and chemical risk were presented, as shown in Figure 4.

**Figure 5:**
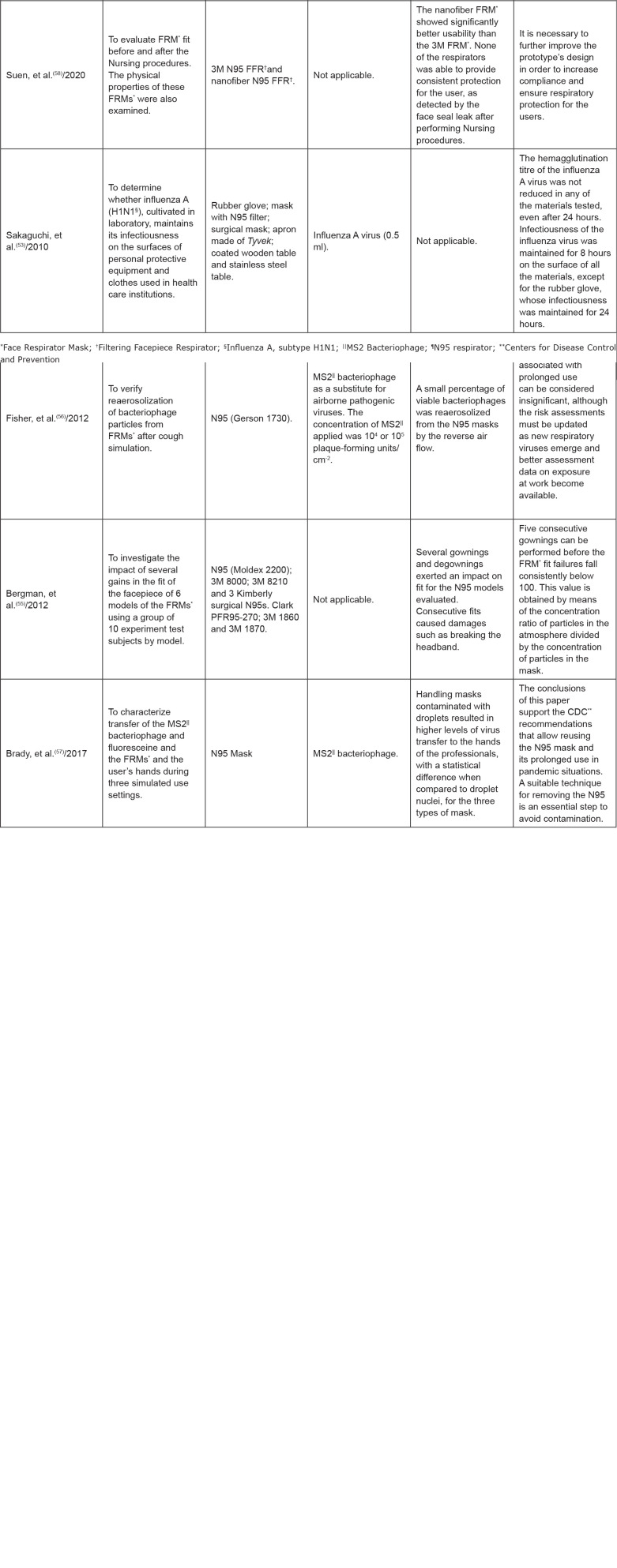
Description of the studies regarding authors, objectives, type of mask, size and type of the microorganisms, effect of reuse on the structure of the masks and recommendation regarding reuse. Ribeirão Preto, SP, Brazil, 2020

The descriptions of the studies regarding authors, objectives, type of mask, size and type of the microorganisms, effect of reuse on the structure of the masks and recommendation regarding reuse are presented in Figure 5.

## Discussion

This study showed the complexity for the effective reprocessing and reuse of the N95 FRMs.

A successful decontamination method must inactivate the virus, not impair performance of the filter, not affect fit of the FRMs, not cause irritation to the user due to residual chemicals, and be easily performed in a timely manner^(56)^.

Regarding reprocessing of the N95 FRMs and equivalent, we noticed that many methods were used for this purpose: ultraviolet germicidal irradiation was used in 14 studies^(27-28,30-31,33-35,37,39-41,45,47,51)^. Among them, ultraviolet light varied from 254 to 302 nanometers, and the doses ranged from 1 to 950 J/cm^2^. The exposure time varied from one to 266 minutes. The authors identified^(41)^ that ultraviolet light administered as a cycle lasting one minute and 30 minutes reduced contamination, but did not meet the decontamination criteria for all places in the FRMs. Other authors showed that the FRMs can be decontaminated and reused up to three times employing ultraviolet light^(45)^. Significant reductions(≥3 log) were observed in the viability of the influenza virus in 12 of 15 models tested and in relation to the straps of 7 of 15 models^(40)^. The authors suggest that decontamination of the N95 mask using ultraviolet can be effective, but it depends on the model, type and material of the FRMs. They also found that ultraviolet light was the only method that did not cause observable physical changes in the FRMs^(22)^. However, only one model passed 20 fit tests and five models did not go through the test^(47)^.

Thus, the use of ultraviolet light is still controversial in terms of decontamination and effectiveness of the FRMs.

As for the use of hydrogen peroxide, we identified eight studies^(27-28,30,41-42,44-46)^. The authors suggest that this method is promising in relation to FRM decontamination, although concerns remain about the residuals left after decontamination^(27)^. However, a research study showed that, in four hours, the hydrogen peroxide levels were reduced below the detection level(0 parts *per* million)^(46)^. The FRMs can be decontaminated and reused up to three times using hydrogen peroxide vapor^(45)^. The decontamination effectiveness of the FRMs was demonstrated with a 31-minute long cycle^(41)^. In addition to that, in the treatment with gaseous hydrogen peroxide, the mean penetration levels were>5% for four of the six FRM models tested^(28)^.

Although promising in relation to the destruction of microorganisms, this method can compromise filtration efficiency of the FRMs.

Regarding the use of vapor methods, four studies used decontamination with autoclave^(38-39,48,50)^. The exposure time varied from 15 to 60 minutes and the temperature from 115 to 121°C. In only one of the studies, immediate-use vapor decontamination was employed^(50)^. It was observed that particle retention was reduced after each autoclave cycle, although the minimum requirements were maintained in the fit test for up to three autoclaving processes^(48)^. In addition to that, a slight elasticity loss was observed in the rubber straps with each autoclave treatment. The masks that went through five processing procedures failed the fit test and presented observable folds^(38)^. It is also noteworthy that some studies used temperatures below 121°C to sterilize the FRMs, being that, in the sterilizing phase, the prescribed temperature for the cycle would be 121 or 134°C, depending on the exposure time^(59)^. It is emphasized that this method caused structural damage that can compromise the effectiveness of the FRMs.

Other studies also used vapor as a resource for decontaminating FRMs. Three of them used a vapor rice cooker^(38-39,49)^, six used microwave-generated vapor^(27-28,31,33-35)^ and one used vapor from a boiling water cup^(51)^. It is emphasized that these methods presented satisfactory results with respect to decontamination of microorganisms; however, they can cause structural damage to the FRMs. In addition to that, these reprocessing methods are not regulated for use in health services.

Regarding dry heat, five studies employed this method^(41,43,45,49,51)^. Temperatures varied from 60 to 100°C and time, from 15 minutes to three hours. Dry heat at 60°C and 70°C for one hour were able to successfully destroy the microorganisms tested and the FRM filtration efficiency was 98%, 98% and 97% after being heated for one, two and three hours, respectively^(43)^. Dry heat at 70°C for 30minutes was not effective in decontaminating bacteriophages^(41)^. A number of researchers showed that, at 70°C, dry heat can be used one or two times without impairing filtration of the FRMs^(45)^, confirming other findings that evidenced filtration efficiency of 96.67%(±0.65) after using dry heat^(51)^.

For effective sterilization of the materials, the oven must be kept closed continuously for 60 minutes with the temperature at 170°C, or for 120 minutes at 160°C. None of the studies used these parameters. Thus, it is not possible to talk about sterilizing the FRMs^(60)^. Therefore, in relation to this method, there are doubts about the real effectiveness of this process in decontaminating the FRMs.

Regarding the use of chemical methods, eight studies were developed. Six used sodium hypochlorite^(27,30,36,38-40,51)^, four^(38-39,45,51)^ tested ethanol and one study used mixed oxidants. Different concentrations and volumes were used, but the odor of chlorine-based solutions remained after decontamination of the FRMs; in addition to that, bleach corroded the metal parts of the FRMs. This result was expected, considering that chlorine is an oxidizing agent.

Regarding filtration efficiency, it was shown that the ethanol- and chlorine-based solutions drastically degraded filtration efficiency to unacceptable levels, 56.33%(±3.03) with ethanol and 73.11%(±7.32) with the chlorine-based solution^(51)^, confirming other findings which showed that decontamination reduced filter quality after using 70% ethanol^(38)^. Ethanol is an intermediate level disinfectant agent and acts on lipid viruses like SARS-CoV-2; however, its action depends on friction, which can explain degradation of the filtration efficiency. It is noteworthy that, in the design of the studies evaluating the chemical methods for decontaminating the FRMs, previous knowledge about the reprocessing methods were not taken into account. It is presumed that exposure of a filter as the one found in the FRMs can be altered when using decontamination liquid products, like ethanol and chlorine.

When analyzing the methods for decontaminating the FRMs, we did not find sufficient evidence to support their reprocessing. We also point out that, in Brazil, any article to be reprocessed must have a validation protocol according to Collegiate Board Resolution RDC2606 of August11^th^,2006, which indicates cleaning, rinsing, drying, packaging, disinfection/sterilization, labeling and conditioning reprocessing phases^(61)^.

In the case of the FRMs, cleaning and rinsing were not performed in the studies analyzed, probably due to the risk of damaging the filter. We also emphasize that, for an article to be subjectable to reprocessing, it must maintain its characteristics, and its efficiency and physical characteristics must be assessed. The reprocessing protocol must also be prepared for each brand and in each of the health institutions, considering the different conditions of the equipment used for the cleaning/disinfection/sterilization procedures.

Another factor to be discussed is the major difficulty in defining decontamination of N95 masks, as determining the microbial load in the different clinical settings and activities is a limiting factor.

Regarding FRM reuse, from the total of studies identified, only seven(21.8%) addressed this topic. A research study^(57)^ showed the transfer of microorganisms from the FRMs to the users’ hands while handling and reusing them.

The health professional must not come into contact with the outer surface of the FRMs, for being considered contaminated. In addition to that, to avoid contamination, it is recommended to pay special attention to the adequate sequence and technique for mask removal after use, holding it by the straps placed on the back of the head^(14)^.

To reuse the FRM, the health professional must inspect it regarding its integrity, including the straps and nose clip that may present changes in their structure which affect fit and seal quality. In addition, the fit test must be performed immediately after placing the FRM to verify proper seal on the user’s face so as to prevent air leakage. To this end, in general, this test is performed by placing both hands on the surface of the mask. The inspection, placement and removal of the mask after use involve its handling, increasing the chance for self-contamination.

The influenzaA virus maintained its ineffectiveness on the surfaces of the surgical mask and of the FRM for at least eight hours^(53)^. Thus, to prevent contamination, it is recommended to pay special attention to the adequate sequence and technique for removing the mask after use^(14)^.

Hand hygiene before and after PPE gowning and degowning and during the assistance provided to limit contamination of the health care environments deserves to be highlighted. In relation to SARS-CoV-2, a study showed that survival time on the human skin is approximately nine hours and increases the risk of viral transmission to other skin surfaces. On the other hand, SARS-CoV-2 was completely inactivated within 15 seconds of exposure to 80%(w/w) ethanol^(62)^.

In the same sense, a study on the infectiousness of the influenza virus in the same PPE identified that it remained active on the surface of the FRMs for at least 8 hours, showing that PPE disposal to prevent cross-infection is an important practice. The researchers point out that reuse of the PPE can be responsible for cross-transmission of the influenza virus and, therefore, it is recommended to discard the mask when it becomes soiled with blood and respiratory secretions, immediately after use^(57)^, and frequent replacement of the PPE for each patient as a preventive measure^(53)^.

Another aspect related to the prolonged use of the FRMs refers to the risk of airborne transmission of particles containing virus, that is, whether they might act as a potential source of exposure risks due to reaerosolization. A research study showed that only a small percentage(≤0.21%) of viable virus was reaerosolized from the tested FRMs by the reverse air flow generated by simulated cough. The viruses applied as aerosols were much more susceptible to reaerosolization than those contaminated with droplets. Thus, the authors point out that the potential threat of reaerosolization, associated with prolonged use of the N95 mask, of most of the respiratory viruses seems insignificant and unlikely to health professionals and patients and that there is a need for studies as new respiratory pathogens emerge^(56)^.

In relation to the research studies that analyzed the potential for contamination by pathogens of the FRMs and their transmission by contact and possibility for reaerosolization, all the studies were conducted in laboratories and, up to date, none of them studied the permanence and ineffectiveness of SARS-CoV-2.

Another concern with reusing N95 masks refers to the damage that multiple placements and removals can cause in their components(such as head straps, strap accessories, adjustable nose tips, etc.), which can adversely affect fit in the user’s face and a proper seal over time^(55)^.

Proper sealing of the FRMs on the user’s face is fundamental for them to maintain adequate protection and comfort. One study showed a progressive decline in the loads generated in the top and bottom straps of the three tested FRM models analyzed over several placement and removal simulations. The largest reduction in the loads occurred within the first 15 minutes of stress, regardless of the mask model, and the magnitude of the load decline depended on the mask model for the upper and lower straps^(54)^.

A research study showed that multiple placements and removals of the FRMs exert an impact on fit in six types of masks analyzed and was associated with the mask model. The data showed that five consecutive placements can be carried out before there is any failure(FF<100)^(55)^.

A study assessed the damage imposed on filter masks over time and estimated their validity period in the clinical practice, showing that, from the fifth day on, all masks were soiled and that folds were observed in more than 80%^(52)^. Internal stains and folds were more common after 12-hour shifts than after 6-hour shifts. It was also identified that 16.17% of the masks were lost on the fifth day and 38.93% after 30 days of use, showing that use of the FRMs must be exclusive for a 12-working-hour shift at the most or, if reuse is really necessary, that the five-day validity period must be respected.

Given the limitation of the evidence found, more research studies are needed to establish the reuse time for the FRMs, especially in real work environments.

Ideally, FRMs should be discarded after each encounter with the patient and after aerosol-generating procedures, when damaged or deformed, when they no longer form an effective seal on the face, when they get wet or visibly soiled, when breathing becomes difficult, as well as when they become contaminated with blood, respiratory or nasal secretions, or other body fluids^(14)^.

For reusing the FRMs, the need for health care institutions to provide a suitable place for storage stands out, preventing their contamination.

Another aspect identified in this research refers to the usability of the FRMs, which is important because discomfort during use can affect compliance. Thus, a study evaluating the physical properties and usability of different FRM brands identified that those produced with nanofiber showed better usability than other materials in terms of facial warmth, breathability, facial pressure, speech intelligibility, itching, difficulty in maintaining the mask in place and comfort level. The nanofiber FRMs were also thinner and lighter and presented slightly higher bacterial filtration efficiency than the other masks evaluated^(58)^.

The studies analyzed allow some recommendations to be listed, such as: 1)the need to train the health professionals working in the care of patients with infectious diseases, 2)the proper technique for placement and removal of the FRMs, as they can be fomites with potential for transmission of pathogens through contact, 3)prevention measures, such as the standard precautions with an emphasis on hand hygiene and measures to limit contamination of health care environments, in order to prevent cross-transmission of microorganisms between health professionals and patients, and 4)reuse is not indicated due to the risk of self-contamination and inadequate sealing.

Thus, as new respiratory pathogens emerge(at increased levels and/or of unknown virulence), there is a need for studies that focus on the possibility for reaerosolization. Future studies assessing the risks of prolonged use for the N95 mask should consider factors such as microbial load, stability of the organism in the environment, performance of the existing engineering controls, and exposure duration.

Finally, there is also a need for studies focused on the improvement of mask designs that favor usability of the FRMs.

We emphasize that more research studies are needed to obtain evidence, especially in real work environments for reusing and reprocessing FRMs, whether or not recommended.

The evidence from this review is indeed timely to the pandemic time of COVID-19 that the world is facing. Reflecting and applying knowledge about reuse and reprocessing of FRMs can contribute to and enrich the health authorities’ decisions. Safety in the health professionals’ work is fundamental against a high-transmissibility pathogen capability like SARS-CoV-2. Adherence to the precautions, especially hand hygiene, correct use of PPE, whether during gowning or degowning, should be strictly followed.

When considering the contributions of this study, some limitations should be listed, as the fact that the studies do not use the FRMs employed in the clinical practice, that none of the studies has carried out the necessary steps for reprocessing validation, as well as the fact that none of studies has used masks contaminated with SARS-CoV-2 virus in the health services. We also point out that, although we have assessed the level of evidence of the articles, we did not assess the methodological quality of the studies included in the review.

## Conclusion

No evidence was found to support safe reprocessing of FRMs. The chemical methods studied should not be used, as they compromise mask integrity. Hydrogen peroxide vapor was listed as an effective method for decontaminating masks and causing less physical damage to them. However, we emphasize that no study conducted all the necessary steps for reprocessing validation. Reuse is contraindicated; however, health institutions perform this practice when they face situations of FRM shortage. A number of studies point out that adequate gowning and hand hygiene before and after removing the mask, as well as proper storage, can prevent mask contamination. In addition to that, mask integrity can be preserved for up to five reuse instances.
